# Socioeconomic differences in the risk of childhood central nervous system tumors in Denmark: a nationwide register-based case–control study

**DOI:** 10.1007/s10552-020-01332-x

**Published:** 2020-08-07

**Authors:** Friederike Erdmann, Ulla Arthur Hvidtfeldt, Mette Sørensen, Ole Raaschou-Nielsen

**Affiliations:** 1 Danish Cancer Society Research Center, Danish Cancer Society, Strandboulevarden 49, 2100 Copenhagen, Denmark; 2 German Childhood Cancer Registry, Institute for Medical Biostatistics, Epidemiology and Informatics (IMBEI), University Medical Center of the Johannes Gutenberg University Mainz, Obere Zahlbacher Str. 69, 55131 Mainz, Germany; 3 Department of Natural Science and Environment, Roskilde University, Universitetsvej 1, P.O. Box 260, 4000 Roskilde, Denmark; 4 Department of Environmental Science, Aarhus University, Frederiksborgvej 399, P.O. Box 358, 4000 Roskilde, Denmark

**Keywords:** Tumors of the central nervous system, Childhood, Childhood cancer, Socioeconomic factors, Socioeconomic status, Denmark, Register-based study

## Abstract

**Purpose:**

Differences in the risk of childhood central nervous system (CNS) tumors by socioeconomic status (SES) may enhance etiologic insights. We conducted a nationwide register-based case–control study to evaluate socioeconomic differences in the risk of childhood CNS tumors in Denmark and examined whether associations varied by different SES measures, time points of assessment, specific tumor types, and age at diagnosis.

**Methods:**

We identified all children born between 1981 and 2013 and diagnosed with a CNS tumor at ages 0–19 years (*n* = 1,273) from the Danish Cancer Registry and sampled four individually matched controls per case (*n*  = 5,086). We used conditional logistic regression models to estimate associations with individual-level and neighborhood-level socioeconomic measures.

**Results:**

We observed elevated risks of ependymoma and embryonal CNS tumors in association with higher parental education (odds ratios (ORs) of 1.6–2.1 for maternal or paternal high education and ependymoma) and higher risk of all tumor types in association with higher maternal income, e.g., OR  1.93; 95% CI 1.05–3.52 for high versus low income for astrocytoma and other gliomas. Associations were often stronger in children diagnosed at ages 5–19 years. We found little evidence for an association with neighborhood SES.

**Conclusion:**

This large nationwide register study with minimal risk of bias showed that having parents with higher educational level and a mother with higher income was associated with a higher risk of childhood CNS tumors. Bias or under-ascertainment of cases among families with low income or basic education is unlikely to explain our findings.

**Electronic supplementary material:**

The online version of this article (10.1007/s10552-020-01332-x) contains supplementary material, which is available to authorized users.

## Introduction

Tumors of the central nervous system (CNS) are the most frequent solid tumors in children, accounting for around 20–25% of all cancers diagnosed in 0–19 year olds in high-income countries [1]. Its two most common subtypes in children, namely pilocytic astrocytoma and medulloblastoma, are rarely seen in adults, whereas the most common adult types, glioblastoma and meningioma, are rarely diagnosed in children [2]. The etiology of childhood CNS tumors is still poorly understood [2, 3] and preventive measures to preclude the disease are lacking. A growing body of research has targeted parental lifestyle factors [4–6], occupational exposures [7, 8], or environmental pollutants [9–12] as possible risk factors but has hitherto provided inconclusive evidence [2, 3, 13]. Certain genetic syndromes, exposure to high-dose ionizing radiation, and high or low birth weight [2, 3, 14, 15] are the only well-established risk factors to date and those explain only a minor proportion (5–10%) of all cases [2, 3]. The early age at diagnosis suggests an important inherited component and that the cancer initiating event might occur before conception, during pregnancy, or in early childhood [2, 16].

Although socioeconomic status (SES) is not an etiological risk factor for disease occurrence per se, it may be associated with risk through mediating factors, including environmental pollutants, parental occupation, or characteristics in relation to pregnancy and parental life style. Knowledge about differences in the risk by socioeconomic group may therefore enhance etiologic insights [17]. To date, only little research has addressed effects of socioeconomic differences on the risk of childhood CNS tumors [18–28] with overall inconsistent findings. Most studies found higher SES being associated with higher risk [18, 21, 23, 24–27], but also null associations [19, 20, 22, 28] and rarely inverse associations were reported [26]. Studies varied substantially by design and SES measures used (including whether the SES indicator was at the individual [18, 21, 24–26] versus neighborhood level [19, 20, 22, 23, 28]) which hinders cross-study comparisons. Moreover, previous investigations suffered largely from methodological limitations including small sample size, lack of subtype specific analyses, single or few SES measures examined and assessed only at a single time point.

The welfare system of a country is obviously important; some countries have large differences in access to and quality of health care services and a relationship with SES is therefore not unexpected but not related to true etiological differences. The Nordic countries are a particular interesting setting for this research question, given that health care is largely free and uniformly accessible together with optimal opportunities for designing nationwide population-based register studies [29] with minimal risk of bias. However, only one regional investigation from Norway is published so far [26], observing that higher family income was associated with a higher risk of ependymomas and embryonal tumors, while a reverse association was seen for astrocytoma [26]. Notably, numbers of subjects in this study were small, and effect estimates statistically imprecise.

To take advantage of the national population-based registries with high-quality and detailed health and socioeconomic data in Denmark, we assessed socioeconomic differences in the risk of CNS tumors in Danish children. We sought to evaluate whether associations varied by different measures of SES, time point of assessment, CNS subtypes, and child’s age at diagnosis. Moreover, we aimed to assess whether demographic and pregnancy-related risk factors mediated associations between SES and childhood CNS tumor risk.

## Material and methods

We conducted a nationwide matched case–control study based on Danish registry data. Denmark has a civil registration system with national population-based administrative registries, [29] such as the Danish Cancer Registry [30], the Central Population Register and several social registries administered by Statistics Denmark [31], and a unique personal identification number (CPR number) used in all registries [29]. Data linkage between those registries provided the basis for our study.

### Study population

We identified cases of first, primary CNS tumors in children aged 0–19 years, born and diagnosed between 1 January 1981 and 31 December 2013 from the Danish Cancer Registry, a nationwide register of all cancers diagnosed in Denmark with excellent quality and high completeness (95–98%) [30, 32]. Four controls per case were sampled randomly by incidence density sampling from the entire childhood population of Denmark using the Central Population Register. Cases and controls were individually matched by sex and date of birth. Cases and controls had to be living in Denmark at date of birth and controls had to be alive and cancer-free at time of diagnosis of the corresponding case to be eligible as control, resulting in a final analytical sample of 1,273 cases and 5,086 controls (*n* = 6,359).

### Classification of CNS tumors

CNS tumors were classified according to the International Classification of Childhood Cancer (ICCC 1st version (i.e., the Birch and Marsden Classification) [33] until 2003 and ICCC 3rd version [34] thereafter) and specific CNS types grouped as follows: Ependymoma (defined by ICCC 1 and ICCC 3 group 3a), astrocytoma and other gliomas (ICCC 1 and ICCC 3 groups 3b and 3d combined), embryonal CNS tumors (defined by ICCC 1 and ICCC 3 group 3c), and other specified and unspecified (ICCC 1 and ICCC 3 groups 3e and 3f combined). With this grouping, we aggregated similar CNS types into groups of larger sample sizes to increase statistical power and to overcome dissimilarities in the two classification versions of ICCC, while keeping distinct diagnostic subtypes separate for meaningful analysis.

### Measures of SES

The theoretical construct of “socioeconomic status” refers to both material and social resources and assets as well as individual’s rank or standing within a social hierarchy of a society [17]. SES is operationalized in numerous ways including measures at the individual-level as well as ecological grouping which indicates the complexity of the multidimensional construct [17, 35]. To address limitations of previous research, we evaluated different indicators of SES including both individual SES measures and neighborhood-based measures, and compared those different SES measures acting at different points in time: at time of conception, during pregnancy, and before diagnosis.

As indicators for the child’s individual-level SES, we used maternal and paternal highest attained education and annual disposable income. The child’s unique CPR number allowed linkage to first-degree relatives via the Danish Civil Registration System considered to be 100% accurate [29]. We obtained annual information on maternal and paternal highest attained education and disposable income from the Danish social registers [36, 37] administered by Statistics Denmark. We categorized the highest educational level into basic [primary and lower secondary education, ≤ 9 years in Denmark], medium [upper secondary including vocational upper secondary education, 10–12 years], and high [> 12 years] education, following the International Standard Classification of Education. Disposable income refers to the annual individual income after tax, interest, and alimony payments and was categorized into five groups based on the sex- and calendar year-specific income distribution (quintiles) of the entire Danish population.

We operationalized three neighborhood SES measures, which reflect the proportion of inhabitants with (i) basic education, (ii) low disposable income level, and (iii) manual profession, respectively, in a given parish. A parish is the smallest geographical unit at which socioeconomic information is available in Denmark. In 2013, parishes (*n* = 2,160) differed in size from 0.12 to 126.2 km^2^ (mean area = 19.9 km^2^) and in population from 26 to 42,251 inhabitants (median = 1,037 inhabitants). We obtained parish codes from the Danish Geodata Agency and socioeconomic data aggregated to the parish level by calendar year from Statistics Denmark. We defined the neighborhood SES measures as follows: proportion of inhabitants aged 30–60 years in a given parish with (i) basic education as the highest attained educational level, (ii) low disposable income level (defined as family disposable income among the lowest quartile of the income distribution of the entire Danish population), and (iii) manual profession (defined as unskilled or semi-skilled profession). As 30–60-year-old individuals of a society determine usually strongest the socioeconomic resources and assets of the respective neighborhood, we focused only on this population group. We assigned five levels of SES for each neighborhood SES measure by calculating quintiles of the respective SES measure proportion distribution across all parishes in Denmark in a given calendar year, weighted by the number of 30–60-year-old inhabitants living in a respective parish. Level 1 indicates highest level of SES, as it reflects the lowest proportions of inhabitations with basic education, low disposable income, or manual profession in a parish, while level 5 stands for the lowest level of SES. We traced residential addresses of the children during childhood and their mothers during pregnancy via the Central Population Register, identified the corresponding parish, and assigned each child the socioeconomic level according to the respective parish at the relevant point in time.

All socioeconomic information was applied to the three time points of interest: at conception, during pregnancy, and before diagnosis (as proxy for the time during childhood, defined as one year before date of diagnosis/index date). In the scenario that one year before diagnosis was before “during pregnancy,” the SES measures corresponded to the situation during pregnancy.

### Demographic and pregnancy-related characteristics

We used information on parental age from the Central Population Register. Data on birthweight were obtained from the Medical Birth Register, which contains mandatory, continuously updated reports on all births in Denmark [38]. Number of siblings included all full and half siblings (defined as having either the same mother or the same father, stillborn children excluded) assessed one year before cancer diagnosis or reference date in controls via the Danish Fertility Database [38].

### Statistical analysis

To assess the correlations between the individual and neighborhood-level SES measures and between time points of assessment, we calculated Spearman’s rank correlation coefficients (*r*_s_).

We fitted conditional logistic regression models to examine the association of socioeconomic measures with the risk of childhood CNS tumors and conducted separate analyses by SES measure, CNS subtype, and time point. The analyses were accounted for sex, age at diagnosis, and calendar time by keeping the individual matching. We further adjusted for maternal or paternal age at child’s birth, respectively, to account for potential confounding. Results were expressed as odds ratios (OR) with two-sided 95% confidence intervals (CI).

We post hoc categorized maternal and paternal income into low, medium, and high by defining the 1st population-based quintile group as “low,” combining the 2nd and 3rd population-based quintile groups to “medium” and combining the 4th and 5th quintile groups to “high” as preliminary analyses had consistently shown similar effect estimates for the now combined groups. As the neighborhood SES measures were only available for the years from 1986 onwards, we had to restrict this analysis to children born in 1986 or later.

We performed the following additional analyses: (i) to evaluate whether associations varied according to age at diagnosis, we conducted analyses by strata of age at diagnosis; (ii) to explore whether associations were accounted for by established demographic and pregnancy-related risk factor [2, 3, 14], we repeated analyses for main findings with further adjustment for birthweight and number of siblings; (iii) to examine the association of SES and childhood CNS tumor risk in the offspring of younger mothers in comparison to older mothers, we conducted stratified analysis by maternal age (younger 30 years of age at child’s birth versus 30 years and older) for our main findings; (iv) finally, we tested the independence of associations seen for individual SES measures by simultaneously fitting models with both individual and neighborhood SES covariates.

All statistical analyses were performed using STATA, version 14.2 [39].

## Results

Of the 1,273 children with a CNS tumor, 426 (33.5%) were diagnosed with astrocytoma and other gliomas, 182 (14.3%) with embryonal CNS tumors, and 99 with ependymoma (7.8%), while 566 (44.5%) were other and unspecified subtypes. About 35% of the cases were diagnosed before the age of 5 years (Table 1). Cases and controls varied only slightly in the distribution of maternal and paternal age and number of siblings, while high birthweight was more prevalent in children with CNS tumors than in control children. Supplementary Material S1 and S2 display the distribution of individual and neighborhood-level socioeconomic measures among cases and controls. The proportion of missing information was overall very low and varied by SES measure and time point of assessment between 5.8 and 0.3% (Material S1 and S2).

**Table 1:** Characteristics of the study population, cases of CNS tumors^a^ in children aged 0–19 years born and diagnosed between 1981 and 2013 in Denmark and matched controls

	**Controls**		**All CNS tumors**		**Ependymoma**		**Astrocytoma and other gliomas**		**Embryonal CNS tumors**		**Other und unspecified**	
	*n*	%	*n*	%	*n*	%	*n*	%	*n*	%	*n*	%
**Sex**												
Boy	2619	51.5	656	51.5	54	54.6	206	48.4	101	55.5	295	52.1
Girl	2467	48.5	617	48.5	45	45.5	220	51.6	81	44.5	271	47.9
**Age at diagnosis/at index date**												
< 1	420	8.3	105	8.3	14	14.1	25	5.9	18	9.9	48	8.5
1–4	1333	26.2	334	26.2	37	37.4	127	29.8	65	35.7	105	18.6
5–9	1430	28.1	358	28.1	21	21.2	124	29.1	66	36.3	147	26.0
10–14	996	19.6	249	19.6	13	13.1	88	20.7	23	12.6	125	22.1
15–19	907	17.8	227	17.8	14	14.4	62	14.6	10	5.5	141	24.9
**Year of birth**												
1981–1990	2084	41.0	521	40.9	47	47.5	195	45.8	67	36.8	212	37.5
1991–2000	2037	40.1	510	40.1	35	35.4	170	39.9	70	38.5	235	41.5
2001–2013	965	19.0	242	19.0	17	17.2	61	14.3	45	24.7	119	21.0
**Year of diagnosis/of index date**												
1981–1990	480	9.4	120	9.4	9	9.1	51	12.0	26	14.3	34	6.0
1991–2000	1763	34.7	441	34.6	46	46.5	168	39.4	59	32.4	168	29.7
2001–2013	2843	55.9	712	55.9	44	44.4	207	48.6	97	53.3	364	64.3
**Maternal age at child’s birth**												
< 25	1145	22.5	267	21.0	22	22.2	97	22.8	38	20.9	110	19.4
25–29	1926	37.9	517	40.6	39	39.4	171	40.1	78	42.9	229	40.5
30–34	1442	28.4	353	27.7	25	25.3	116	27.2	43	23.6	169	29.9
≥ 35	572	11.3	136	10.7	13	13.1	42	9.9	23	12.6	58	10.3
**Paternal age at child’s birth**												
< 25	519	10.3	133	10.6	8	8.1	55	13.0	19	10.6	51	9.1
25–29	1616	32.0	407	32.3	37	37.4	133	31.5	62	34.4	175	31.3
30–34	1688	33.4	420	33.3	35	35.4	128	30.3	63	35.0	194	34.7
≥ 35	1225	24.3	300	23.8	19	19.2	106	25.1	36	20.0	139	24.9
**Birth weight (g)**												
< 2500	244	4.9	56	4.5	3	3.2	20	4.7	15	8.4	18	3.3
2500–3999	3916	77.8	942	75.4	74	77.9	317	74.8	132	73.7	419	75.9
≥ 4000	875	17.4	252	20.2	18	19.0	87	20.5	32	17.9	115	20.8
**Number of siblings**^b^												
0	788	15.5	219	17.2	26	26.3	70	16.4	38	20.9	85	15.0
1	2225	43.8	565	44.4	43	43.4	178	41.8	96	52.8	248	43.8
2	1324	26.0	331	26.0	21	21.2	124	29.1	33	18.1	153	27.0
≥ 3	749	14.7	158	12.4	9	9.1	54	12.7	15	8.2	80	14.1

Missing information: maternal age: 0.02%; paternal age: 0.80%; birth weight: 1.16%; remaining characteristics have complete information

^a^Classified by the International Classification of Childhood Cancer (ICCC), up to 2003 by Birch & Marsden (first edition) and from 2003 onwards by ICCC 3rd version. Grouped as follows: Ependymoma (defined by ICCC 1 and ICCC 3 group 3a), astrocytoma and other gliomas (ICCC 1 and ICCC 3 groups 3b and 3d combined), embryonal CNS tumors (defined by ICCC 1 and ICCC 3 group 3c) and other specified and unspecified (ICCC 1 and ICCC 3 groups 3e and 3f combined)

^b^Number of full and half siblings (defined as having the same mother or father) assed 1 year before diagnosis. In the scenario, that one year before diagnosis was before “during pregnancy”, the number of siblings corresponds to the situation during pregnancy

We found that level of education was strongly correlated between time points of assessment (Supplementary Material S3). Similarly, maternal and paternal levels of income, respectively, were strongly correlated between time of conception and during pregnancy but only moderately between time before diagnosis and at conception or during pregnancy*.* Individual SES measures were only weakly correlated with neighborhood SES measures (Supplementary Material S4).

### Individual SES measures

#### Maternal and paternal education

Table 2 shows that the risk of childhood CNS tumors overall was slightly elevated for children of parents with higher educational level, displaying, e.g., an OR of 1.18 (95% CI 0.98–1.42) in association with maternal high educational level at time before diagnosis. Analyses by CNS tumor type revealed more distinct, although sometimes imprecise, associations with specific tumor types, often most pronounced in children diagnosed at ages 5–19 years compared to children aged 0–4 years at diagnosis and compared to the full sample.

**Table 2:** Association^a^ between maternal and paternal highest attained education^b^ and risk of CNS tumors in children aged 0–19 years at diagnosis and children aged 5–19 years at diagnosis, by CNS tumor type^c^ and time of assessment

Aged 0–19 years at diagnosis										
	**All CNS tumors**		**Ependymoma**		**Astrocytoma and other gliomas**		**Embryonal CNS tumors**		**Other specified and unspecified**	
	*n* cases	OR (95% CI)	*n* cases	OR (95% CI)	*n* cases	OR (95% CI)	*n* cases	OR (95% CI)	*n* cases	OR (95% CI)
**Maternal education**										
*At conception*										
Basic	321	1.0	22	1.0	117	1.0	41	1.0	141	1.0
Medium	556	0.94 (0.80–1.11)	48	1.22 (0.66–2.26)	197	0.87 (0.66–1.14)	80	1.28 (0.83–1.97)	231	0.86 (0.67–1.10)
High	334	1.10 (0.90–1.33)	24	1.59 (0.77–3.28)	91	0.79 (0.56–1.11)	54	1.46 (0.87–2.46)	165	1.18 (0.89–1.58)
*During pregnancy*										
Basic	307	1.0	22	1.0	115	1.0	37	1.0	133	1.0
Medium	556	0.97 (0.83–1.14)	43	1.09 (0.58–2.03)	195	0.87 (0.66–1.14)	82	1.39 (0.90–2.16)	236	0.93 (0.73–1.19)
High	372	1.12 (0.93–1.36)	31	1.77 (0.89–3.53)	103	0.79 (0.57–1.10)	58	1.50 (0.90–2.50)	180	1.22 (0.93–1.61)
*Before diagnosis*										
Basic	249	1.0	18	1.0	96	1.0	30	1.0	105	1.0
Medium	556	1.00 (0.84–1.19)	43	1.09 (0.56–2.10)	194	0.88 (0.66–1.17)	82	1.59 (0.99–2.54)	237	0.94 (0.72–1.22)
High	439	1.18 (0.98–1.42)	35	1.60 (0.80–3.19)	126	0.86 (0.63–1.19)	66	1.84 (1.09–3.12)	212	1.23 (0.93–1.62)
**Paternal education**										
*At conception*										
Basic	263	1.0	14	1.0	89	1.0	43	1.0	117	1.0
Medium	680	1.08 (0.92–1.28)	64	2.02 (1.05–3.88)	238	1.16 (0.87–1.54)	86	0.96 (0.63–1.46)	292	0.97 (0.76–1.23)
High	263	1.10 (0.90–1.34)	17	2.13 (0.97–4.70)	75	0.94 (0.66–1.36)	46	1.31 (0.79–2.17)	125	1.04 (0.78–1.39)
*During pregnancy*										
Basic	262	1.0	15	1.0	88	1.0	42	1.0	117	1.0
Medium	671	1.05 (0.89–1.23)	62	1.74 (0.93–3.27)	238	1.17 (0.88–1.56)	85	0.94 (0.62–1.44)	286	0.92 (0.72–1.17)
High	288	1.10 (0.91–1.34)	19	2.05 (0.96–4.34)	82	0.96 (0.68–1.37)	48	1.29 (0.78–2.12)	139	1.05 (0.79–1.39)
*Before diagnosis*										
Basic	246	1.0	14	1.0	84	1.0	41	1.0	107	1.0
Medium	640	0.99 (0.84–1.16)	61	1.57 (0.83–2.97)	224	1.03 (0.78–1.38)	80	0.83 (0.55–1.28)	275	0.92 (0.72–1.18)
High	341	1.13 (0.94–1.37)	22	1.78 (0.84–3.74)	103	1.03 (0.73–1.44)	54	1.20 (0.74–1.95)	162	1.11 (0.84–1.46)

Aged 5–19 years at diagnosis										
	All CNS tumors		Ependymoma		Astrocytoma and other gliomas		Embryonal CNS tumors		Other specified and unspecified	
	*n* cases	OR (95% CI)	*n* cases	OR (95% CI)	*n* cases	OR (95% CI)	*n* cases	OR (95% CI)	*n* cases	OR (95% CI)
**Maternal education**										
*At conception*										
Basic	220	1.0	10	1.0	74	1.0	24	1.0	112	1.0
Medium	365	0.94 (0.77–1.14)	25	1.30 (0.56–3.02)	136	0.98 (0.70–1.37)	40	1.16 (0.66–2.06)	164	0.81 (0.61–1.08)
High	206	1.11 (0.87–1.40)	11	1.54 (0.54–4.43)	50	0.74 (0.48–1.15)	31	1.82 (0.93–3.53)	114	1.18 (0.85–1.64)
*During pregnancy*										
Basic	208	1.0	10	1.0	72	1.0	21	1.0	105	1.0
Medium	371	1.0 (0.82–1.21)	25	1.26 (0.54–2.92)	135	1.01 (0.72–1.41)	42	1.36 (0.76–2.43)	169	0.88 (0.66–1.16)
High	228	1.17 (0.92–1.47)	13	1.63 (0.58–4.52)	57	0.80 (0.52–1.22)	33	1.96 (1.01–3.83)	125	1.23 (0.89–1.70)
*Before diagnosis*										
Basic	153	1.0	6	1.0	54	1.0	16	1.0	77	1.0
Medium	378	1.06 (0.86–1.31)	26	1.51 (0.57–4.02)	136	1.09 (0.76–1.57)	41	1.47 (0.78–2.78)	175	0.92 (0.68–1.25)
High	285	1.24 (0.98–1.57)	16	1.68 (0.58–4.89)	77	0.95 (0.63–1.43)	40	2.45 (1.22–4.92)	152	1.22 (0.88–1.68)
**Paternal education**										
*At conception*										
Basic	177	1.0	5	1.0	58	1.0	24	1.0	90	1.0
Medium	442	1.02 (0.84–1.25)	31	2.30 (0.80–6.60)	153	1.11 (0.78–1.58)	46	1.00 (0.56–1.78)	212	0.91 (0.69–1.21)
High	168	1.09 (0.85–1.39)	10	3.65 (1.07–12.50)	45	0.87 (0.55–1.38)	27	1.68 (0.84–3.36)	86	1.00 (0.71–1.41)
*During pregnancy*										
Basic	174	1.0	5	1.0	56	1.0	23	1.0	90	1.0
Medium	441	1.02 (0.83–1.24)	30	2.10 (0.73–6.02)	154	1.13 (0.79–1.61)	45	1.03 (0.58–1.84)	212	0.89 (0.68–1.17)
High	183	1.09 (0.86–1.39)	11	3.82 (1.15–12.66)	50	0.90 (0.58–1.41)	29	1.73 (0.88–3.41)	93	0.98 (0.70–1.37)
*Before diagnosis*										
Basic	160	1.0	5	1.0	53	1.0	22	1.0	80	1.0
Medium	419	0.94 (0.77–1.16)	30	1.74 (0.62–4.89)	144	0.94 (0.66–1.36)	41	0.83 (0.46–1.48)	204	0.91 (0.69–1.22)
High	224	1.09 (0.87–1.38)	12	2.51 (0.78–8.03)	65	0.92 (0.61–1.41)	34	1.44 (0.75–2.74)	113	1.05 (0.76–1.46)

^a^Conditional logistic regression analyses [odds ratio (and 95% confidence interval)] adjusted for maternal or paternal age at child’s birth, respectively, modeled as continuous variable. Accounted for sex, age at diagnosis, and calendar time by design

^b^Categorized according to the highest attained level (basic [primary and lower secondary education, ≤ 9 years]; medium [upper secondary including vocational upper secondary education, 10–12 years]; higher [> 12 years]). Missing information: maternal education at conception: 4.5%; maternal education during pregnancy: 2.6%; maternal education before diagnosis: 2.1%; paternal education at conception: 5.5%; paternal education during pregnancy: 4.1%; paternal education before diagnosis: 3.3%

^c^Classified by the International Classification of Childhood Cancer (ICCC), up to 2003 by Birch & Marsden (first edition) and from 2003 onwards by ICCC 3rd version. Grouped as follows: Ependymoma (defined by ICCC 1 and ICCC 3 group 3a), astrocytoma and other gliomas (ICCC 1 and ICCC 3 groups 3b and 3d combined), embryonal CNS tumors (defined by ICCC 1 and ICCC 3 group 3c), and other specified and unspecified (ICCC 1 and ICCC 3 groups 3e and 3f combined)

High maternal and paternal levels of education were associated with ORs ranging from 1.6 to 2.1 for ependymoma across the different time points of assessment. Associations with paternal education were particular strong in children diagnosed at ages 5–19 years, with the ependymoma risk being increased almost fourfold for high paternal education at conception and during pregnancy (OR during pregnancy = 3.82; 95% CI 1.15–12.66). Also the risk of embryonal CNS tumors was associated with parental level of education. In particular, higher level of maternal education was associated with an increased risk of embryonal CNS tumors in the offspring, most evident for the time before diagnosis and in children diagnosed at ages 5–19 year with an OR of 2.45 (95% CI 1.22–4.92).

Effect estimates for astrocytomas and other gliomas suggested a tendency of a weak inverse association with maternal education.

Overall, risk patterns and effect estimates did not differ markedly between the three different time points under study.

#### Maternal and paternal disposable income

Maternal high and often medium level of disposable income was consistently associated with an increased risk of CNS tumors overall and across individual tumor types (Table 3). Associations were most evident at time before diagnosis and most marked for the risk of astrocytoma and other gliomas (OR before diagnosis = 1.93; 95% CI 1.05–3.52). Patterns and effect sizes were generally less consistent between time points than seen for parental education. An exception is the group of embryonal CNS tumors for which the effect size of estimates were similar between time points, with ORs for high or medium income level ranging from 1.64 to 1.92. We found stronger associations in children diagnosed at ages 5–19 years across all tumor types, most distinct for the risk of ependymoma and embryonal CNS tumors (Supplementary Material S5).

**Table 3:** Association^a^ between maternal and paternal disposable income^b^ and risk of CNS tumors in children aged 0–19 years at diagnosis, by CNS tumor type^c^ and time of assessment

	**All CNS tumors**		**Ependymoma**		**Astrocytoma and other gliomas**		**Embryonal CNS tumors**		**Other specified and unspecified**	
	*n* cases	OR (95% CI)	*n* cases	OR (95% CI)	*n* cases	OR (95% CI)	*n* cases	OR (95% CI)	*n* cases	OR (95% CI)
**Maternal income**										
*At conception*										
Low	115	1.0	11	1.0	36	1.0	12	1.0	56	1.0
Medium	474	1.22 (0.97–1.53)	30	0.83 (0.37–1.83)	169	1.30 (0.87–1.95)	77	1.88 (0.96–3.66)	198	1.10 (0.79–1.53)
High	674	1.28 (1.02–1.62)	57	1.23 (0.57–2.68)	219	1.20 (0.80–1.80)	91	1.92 (0.97–3.80)	307	1.23 (0.88–1.71)
*During pregnancy*										
Low	104	1.0	10	1.0	35	1.0	11	1.0	48	1.0
Medium	481	1.13 (0.89–1.44)	35	0.84 (0.38–1.87)	167	1.08 (0.71–1.62)	79	1.90 (0.95–3.80)	200	1.06 (0.75–1.52)
High	681	1.24 (0.97–1.57)	53	1.05 (0.48–2.29)	224	1.10 (0.73–1.65)	90	1.64 (0.81–3.31)	314	1.29 (0.91–1.84)
*Before diagnosis*										
Low	52	1.0	5	1.0	13	1.0	10	1.0	24	1.0
Medium	378	1.58 (1.15–2.17)	32	1.59 (0.57–4.45)	137	2.27 (1.22–4.19)	51	1.27 (0.60–2.67)	158	1.35 (0.84–2.17)
High	829	1.53 (1.12–2.08)	60	1.50 (0.55–4.11)	273	1.93 (1.05–3.52)	119	1.77 (0.85–3.68)	377	1.27 (0.80–2.02)
**Paternal income**										
*At conception*										
Low	123	1.0	11	1.0	49	1.0	12	1.0	51	1.0
Medium	576	1.04 (0.84–1.30)	52	1.26 (0.61–2.61)	188	1.02 (0.72–1.44)	89	1.43 (0.74–2.76)	247	0.94 (0.67–1.32)
High	553	0.94 (0.76–1.17)	35	0.84 (0.39–1.79)	185	0.92 (0.65–1.31)	79	1.29 (0.66–2.50)	254	0.89 (0.64–1.25)
*During pregnancy*										
Low	124	1.0	9	1.0	48	1.0	12	1.0	55	1.0
Medium	556	0.98 (0.79–1.22)	52	1.40 (0.63–3.11)	183	0.90 (0.63–1.29)	81	1.57 (0.81–3.06)	240	0.86 (0.62–1.20)
High	573	0.90 (0.73–1.12)	37	0.97 (0.43–2.20)	191	0.84 (0.59–1.20)	87	1.60 (0.82–3.10)	258	0.80 (0.58–1.12)
*Before diagnosis*										
Low	93	1.0	6	1.0	37	1.0	12	1.0	38	1.0
Medium	472	1.21 (0.94–1.55)	43	1.70 (0.68–4.28)	165	1.13 (0.76–1.70)	72	1.17 (0.59–2.33)	192	1.21 (0.82–1.78)
High	683	1.12 (0.88–1.43)	49	1.53 (0.62–3.75)	217	0.99 (0.67–1.48)	96	1.13 (0.57–2.21)	321	1.17 (0.80–1.70)

^a^Conditional logistic regression analyses [odds ratio (and 95% confidence interval)] adjusted for maternal or paternal age at child’s birth, respectively, modeled as continuous variable. Accounted for sex, age at diagnosis, and calendar time by design

^b^Refers to the annual individual income after tax, interest, and alimony payments, categorized into *low, medium, and high* based on the income quintiles of the entire Danish population by calendar year and sex (1st quintile: low, 2nd and 3rd quintiles: medium, 4th and 5th quintiles: high). Missing information: maternal income at conception: 0.5%; maternal income during pregnancy: 0.3%; maternal income before diagnosis: 1.0%; paternal income at conception: 1.2%; paternal income during pregnancy: 1.1%; paternal income before diagnosis: 2.1%

^c^Classified by the International Classification of Childhood Cancer, up to 2003 by Birch & Marsden (first edition) and from 2003 onwards by ICCC 3. Grouped as follows: Ependymoma (defined by ICCC 1 and ICCC 3 group 3a), astrocytoma and other gliomas (ICCC 1 and ICCC 3 groups 3b and 3d combined), embryonal CNS tumors (defined by ICCC 1 and ICCC 3 group 3c), and other specified and unspecified (ICCC 1 and ICCC 3 groups 3e and 3f combined)

Patterns for the effect of paternal income were inconclusive. Although ORs for paternal medium and high level of income were elevated for the risk of embryonal CNS tumors at conception and during pregnancy (OR paternal high income during pregnancy = 1.60; 95% CI 0.82–3.10) and for the risk of ependymoma at time before diagnosis, no consistent risk pattern emerged (Table 3).

### Neighborhood SES measures

We noted elevated ORs for the risk of ependymoma in association with living in a neighborhood with lower proportion of inhabitants with basic education or low income (Table 4). ORs were also increased for the risk of embryonal tumors in association with higher neighborhood SES based on the manual profession quintiles. However, no overall trend or other systematic risk patterns were evident for any of the neighborhood SES measures.

**Table 4:** Association^a^ between measures of neighborhood socioeconomic status ^b^ and risk of CNS tumors in children aged 0–19 years at diagnosis, by CNS tumor type^c^ and time of assessment

	**All CNS tumors**		**Ependymoma**		**Astrocytoma and other gliomas**		**Embryonal CNS tumors**		**Other specified and unspecified**	
	*n* cases	OR (95% CI)	*n* cases	OR (95% CI)	*n* cases	OR (95% CI)	*n* cases	OR (95% CI)	*n* cases	OR (95% CI)
**Neighborhood basic education**										
*At conception*										
5 (low SES)	193	1.0	13	1.0	55	1.0	27	1.0	98	1.0
4	190	1.01 (0.81–1.27)	17	1.24 (0.55–2.78)	65	1.38 (0.92–2.06)	23	0.79 (0.42–1.49)	85	0.85 (0.61–1.18)
3	199	1.15 (0.92–1.43)	17	1.62 (0.71–3.70)	69	1.44 (0.97–2.14)	29	1.26 (0.69–2.27)	84	0.89 (0.64–1.24)
2	196	1.10 (0.88–1.37)	14	1.09 (0.46–2.60)	55	1.10 (0.72–1.67)	37	1.29 (0.72–2.29)	90	1.02 (0.74–1.41)
1 (high SES)	199	1.12 (0.89–1.41)	14	1.63 (0.70–3.80)	62	1.21 (0.80–1.81)	28	1.12 (0.62–2.03)	95	0.99 (0.71–1.39)
*During pregnancy*										
5	199	1.0	14	1.0	60	1.0	31	1.0	94	1.0
4	203	1.08 (0.87–1.35)	16	1.30 (0.58–2.94)	70	1.37 (0.93–2.02)	23	0.74 (0.40–1.37)	94	0.99 (0.72–1.36)
3	198	1.12 (0.90–1.39)	17	1.72 (0.75–3.94)	65	1.31 (0.88–1.93)	27	1.04 (0.58–1.87)	89	0.95 (0.69–1.31)
2	205	1.10 (0.88–1.37)	14	0.98 (0.42–2.28)	65	1.20 (0.81–1.77)	39	1.16 (0.67–2.02)	87	1.02 (0.73–1.42)
1	198	1.10 (0.87–1.37)	16	1.87 (0.83–4.21)	61	1.15 (0.77–1.72)	25	0.94 (0.52–1.71)	96	1.01 (0.72–1.40)
*Before diagnosis*										
5	233	1.0	15	1.0	65	1.0	34	1.0	119	1.0
4	202	0.94 (0.76–1.17)	19	1.50 (0.69–3.26)	72	1.19 (0.82–1.72)	23	0.66 (0.36–1.18)	88	0.82 (0.60–1.13)
3	198	0.87 (0.71–1.08)	15	1.14 (0.50–2.59)	69	1.12 (0.77–1.64)	27	0.82 (0.47–1.45)	87	0.72 (0.53–0.98)
2	191	0.88 (0.71–1.09)	15	1.10 (0.50–2.50)	56	0.92 (0.62–1.38)	37	0.98 (0.57–1.67)	83	0.78 (0.57–1.07)
1	202	0.96 (0.78–1.19)	12	1.32 (0.56–3.09)	67	1.13 (0.77–1.65)	30	1.03 (0.59–1.81)	93	0.80 (0.59–1.10)
**Neighborhood low income**										
*At conception*										
5 (low SES)	240	1.0	10	1.0	76	1.0	40	1.0	114	1.0
4	212	1.07 (0.87–1.32)	21	2.34 (1.00–5.49)	65	1.02 (0.70–1.48)	27	0.82 (0.47–1.42)	99	1.10 (0.81–1.48)
3	185	1.01 (0.81–1.25)	17	2.36 (0.98–5.68)	65	1.05 (0.72–1.52)	27	0.83 (0.48–1.44)	76	0.92 (0.66–1.27)
2	161	0.91 (0.73–1.14)	14	1.72 (0.70–4.21)	46	0.87 (0.58–1.31)	18	0.67 (0.36–1.23)	83	0.96 (0.70–1.31)
1 (high SES)	179	1.10 (0.88–1.37)	13	1.52 (0.62–3.73)	54	1.02 (0.69–1.51)	32	1.41 (0.83–2.41)	80	1.03 (0.75–1.43)
*During pregnancy*										
5	237	1.0	7	1.0	74	1.0	40	1.0	116	1.0
4	224	1.16 (0.94–1.42)	19	3.33 (1.29–8.58)	75	1.34 (0.93–1.94)	27	0.78 (0.45–1.37)	103	1.06 (0.78–1.43)
3	196	1.07 (0.87–1.33)	21	5.31 (2.08–13.55)	67	1.20 (0.83–1.73)	29	0.85 (0.50–1.47)	79	0.86 (0.63–1.18)
2	166	0.91 (0.73–1.14)	14	2.37 (0.88–6.40)	48	0.95 (0.64–1.43)	17	0.59 (0.32–1.11)	87	0.91 (0.66–1.23)
1	180	1.11 (0.89–1.38)	16	2.63 (1.02–6.79)	57	1.17 (0.79–1.73)	32	1.38 (0.80–2.40)	75	0.91 (0.66–1.26)
*Before diagnosis*										
5	194	1.0	8	1.0	67	1.0	26	1.0	93	1.0
4	214	1.13 (0.91–1.41)	18	2.35 (0.94–5.89)	63	1.01(0.67–1.47)	30	1.12 (0.62–2.01)	103	1.14 (0.83–1.57)
3	208	0.97 (0.78–1.21)	20	3.44 (1.35–8.76)	71	1.04 (0.71–1.51)	30	0.95 (0.53–1.72)	87	0.78 (0.56–1.09)
2	191	0.91 (0.73–1.14)	16	1.95 (0.75–5.07)	53	0.90 (0.60–1.34)	32	1.03 (0.58–1.85)	90	0.82 (0.60–1.14)
1	219	0.98 (0.79–1.22)	14	1.49 (0.59–3.77)	75	1.07 (0.73–1.55)	33	1.34 (0.75–2.38)	97	0.82 (0.60–1.13)
**Neighborhood manual profession**										
*At conception*										
5 (low SES)	161	1.0	14	1.0	48	1.0	16	1.0	83	1.0
4	206	1.33 (1.06–1.68)	22	1.40 (0.67–2.6)	63	1.21 (0.80–1.85)	37	3.39 (1.75–6.54)	84	1.07 (0.76–1.49)
3	185	1.18 (0.93–1.49)	15	1.04 (0.47–2.32)	59	1.27 (0.83–1.95)	24	1.78 (0.89–3.58)	87	1.05 (0.75–1.47)
2	194	1.22 (0.97–1.54)	11	1.01 (0.42–2.44)	59	1.13 (0.74–1.73)	33	2.58 (1.33–5.02)	91	1.07 (0.77–1.50)
1 (high SES)	231	1.32 (1.05–1.65)	13	1.09 (0.48–2.49)	77	1.31 (0.88–1.96)	34	2.04 (1.08–3.85)	107	1.21 (0.87–1.68)
*During pregnancy*										
5	178	1.0	16	1.0	56	1.0	21	1.0	85	1.0
4	217	1.30 (1.04–1.63)	21	1.22 (0.59–2.53)	69	1.25 (0.84–1.85)	32	2.21 (1.19–4.11)	95	1.17 (0.84–1.62)
3	187	1.10 (0.87–1.38)	14	0.88 (0.41–1.93)	58	1.04 (0.70–1.57)	29	1.48 (0.79–2.77)	86	1.10 (0.79–1.54)
2	189	1.10 (0.88–1.39)	11	0.93 (0.39–2.21)	60	1.05 (0.70–1.58)	30	1.76 (0.94–3.28)	88	1.02 (0.73–1.43)
1	232	1.23 (0.99–1.52)	15	1.02 (0.47–2.21)	78	1.27 (0.86–1.87)	33	1.57 (0.87–2.84)	106	1.14 (0.83–1.57)
*Before diagnosis*										
5	212	1.0	25	1.0	63	1.0	29	1.0	95	1.0
4	225	1.16 (0.94–1.43)	15	0.73 (0.35–1.52)	78	1.29 (0.88–1.87)	35	1.58 (0.90–2.76)	97	1.08 (0.79–1.49)
3	220	1.11 (0.90–1.38)	14	0.71 (0.34–1.47)	65	1.03 (0.70–1.51)	31	1.26 (0.72–2.22)	110	1.26 (0.92–1.72)
2	179	0.99 (0.80–1.24)	11	0.66 (0.30–1.44)	56	0.90 (0.61–1.34)	30	1.33 (0.75–2.37)	82	1.07 (0.77–1.49)
1	190	1.05 (0.84–1.31)	11	0.69 (0.31–1.52)	67	1.17 (0.80–1.73)	26	1.06 (0.59–1.88)	86	1.05 (0.76–1.47)

^a^Conditional logistic regression analyses [odds ratio (and 95% confidence interval)] adjusted for maternal age at child’s birth, modeled as continuous variable. Accounted for sex, age at diagnosis, and calendar time by design

^b^Based on the proportions of inhabitants aged 30–60 years with (i) basic highest attained educational level, (ii) low disposable income, and (iii) manual profession in a given parish. Levels of SES are consecutively numbered; level 1 indicates highest SES, while level 5 stands for lowest SES. Missing information: at conception: 5.8%; during pregnancy: 3.2%; before diagnosis: 2.2%

^c^Classified by the International Classification of Childhood Cancer, up to 2003 by Birch & Marsden (first edition) and from 2003 onwards by ICCC 3. Grouped as follows: Ependymoma (defined by ICCC 1 and ICCC 3 group 3a), astrocytoma and other gliomas (ICCC 1 and ICCC 3 groups 3b and 3d combined), embryonal CNS tumors (defined by ICCC 1 and ICCC 3 group 3c), and other specified and unspecified (ICCC 1 and ICCC 3 groups 3e and 3f combined)

### Independence of associations and additional analyses

As illustrated in Table 5, models adjusted for the effect of birthweight and number of siblings provided similar results to those of the main analysis (Tables 3 and 4). Adjusting the association between parental level of education and income and risk of CNS tumors for measures of neighborhood SES did similarly not affect the overall risk pattern (Table 6). Only effect estimates of the association of maternal income level and ependymoma risk were attenuated towards the null, while other effect estimates did not change appreciably and some associations became stronger. However, confidence intervals were wide.

**Table 5:** Association^a^ between maternal and paternal highest attained education^b^ and disposable income^c^ at time before diagnosis and risk of CNS tumors in children diagnosed at ages 0–19 years, accounted for demographic and pregnancy-related risk factors

	**All CNS tumors**	**Ependymoma**	**Astrocytoma and other gliomas**	**Embryonal CNS tumors**	**Other specified and unspecified**
	OR (95% CI)	OR (95% CI)	OR (95% CI)	OR (95% CI)	OR (95% CI)
**Maternal education**					
Basic	1.0	1.0	1.0	1.0	1.0
Medium	0.98 (0.82–1.16)	0.99(0.50–1.97)	0.88 (0.66–1.18)	1.54 (0.95–2.51)	0.92 (0.70–1.20)
High	1.13 (0.93–1.36)	1.46 (0.72–2.96)	0.84 (0.61–1.17)	1.74 (1.01–3.03)	1.18 (0.89–1.58)
**Paternal education**					
Basic	1.0	1.0	1.0	1.0	1.0
Medium	0.97 (0.82–1.15)	1.64 (0.85–3.16)	1.05 (0.78–1.41)	0.79 (0.51–1.22)	0.88 (0.68–1.13)
High	1.10 (0.91–1.33)	1.77 (0.82–3.80)	1.04 (0.74–1.47)	1.13 (0.69–1.85)	1.04 (0.78–1.38)
**Maternal income**					
Low	1.0	1.0	1.0	1.0	1.0
Medium	1.57 (1.14–2.17)	1.60 (0.56–4.56)	2.29 (1.23–4.26)	1.18 (0.56–2.49)	1.38 (0.85–2.24)
High	1.50 (1.09–2.05)	1.51 (0.54–4.27)	1.94 (1.06–3.56)	1.59 (0.76–3.31)	1.27 (0.80–2.04)
**Paternal income**					
Low	1.0	1.0	1.0	1.0	1.0
Medium	1.18 (0.92–1.52)	1.36 (0.54–3.46)	1.14 (0.76–1.71)	1.11 (0.56–2.23)	1.19 (0.81–1.77)
High	1.09 (0.85–1.39)	1.32 (0.53–3.32)	1.01 (0.67–1.51)	1.04 (0.52–2.06)	1.14 (0.77–1.67)

^a^Conditional logistic regression analyses [odds ratio (and 95% confidence interval)] adjusted for maternal or paternal age at child’s birth, respectively (modeled as continuous variable), birthweight, and number of siblings. Accounted for sex, age at diagnosis, and calendar time by design

^b^Categorized according to the highest attained level [basic (primary and lower secondary education, ≤ 9); medium (upper secondary including vocational upper secondary education, 10–12 years); higher (> 12 years)]

^c^Refers to the annual individual income after tax, interest, and alimony payments, categorized into *low, medium, and high* based on the income quintiles of the entire Danish population by calendar year and sex (1st quintile: low, 2nd and 3rd quintiles: medium, 4th and 5th quintiles: high)

**Table 6:** Association^a^ between maternal and paternal highest attained education^b^ and disposable income^c^ at time before diagnosis and risk of CNS tumors in children diagnosed at ages 0–19 years, accounted for neighborhood socioeconomic status

	**All CNS tumors**	**Ependymoma**	**Astrocytoma and other gliomas**	**Embryonal CNS tumors**	**Other specified and unspecified**
	OR (95% CI)	OR (95% CI)	OR (95% CI)	OR (95% CI)	OR (95% CI)
**Maternal education**					
Basic	1.0	1.0	1.0	1.0	1.0
Medium	1.04 (0.87–1.25)	1.06 (0.51–2.21)	0.90 (0.67–1.22)	1.61 (0.98–2.63)	1.01 (0.77–1.33)
High	1.22 (1.00–1.48)	1.44 (0.65–3.17)	0.84 (0.60–1.18)	1.99 (1.14–3.48)	1.36 (1.01–1.82)
**Paternal education**					
Basic	1.0	1.0	1.0	1.0	1.0
Medium	1.00 (0.84–1.18)	1.72 (0.83–3.56)	1.04 (0.77–1.39)	0.83 (0.53–1.29)	0.95 (0.73–1.22)
High	1.15 (0.94–1.41)	2.23 (0.93–5.34)	1.01 (0.71–1.44)	1.24 (0.74–2.06)	1.15 (0.86–1.54)
**Maternal income**					
Low	1.0	1.0	1.0	1.0	1.0
Medium	1.46 (1.04–2.04)	1.15 (0.38–3.46)	2.11 (1.10–4.03)	1.05 (0.47–2.37)	1.31 (0.789–2.17)
High	1.43 (1.03–1.98)	1.08 (0.36–3.20)	1.79 (0.95–3.38)	1.62 (0.73–3.61)	1.26 (0.77–2.05)
**Paternal income**					
Low	1.0	1.0	1.0	1.0	1.0
Medium	1.16 (0.89–1.50)	2.12 (0.69–6.54)	1.06 (0.69–1.61)	0.99 (0.49–2.01)	1.17 (0.79–1.75)
High	1.08 (0.84–1.40)	2.18 (0.72–6.59)	0.91 (0.60–1.38)	0.92 (0.46–1.86)	1.17 (0.79–1.74)

^a^Conditional logistic regression analyses [odds ratio (and 95% confidence interval)] adjusted for maternal or paternal age at child’s birth, respectively (modeled as continuous variable), and three measures of neighborhood SES which reflect the proportions of inhabitants aged 30–60 years with (i) basic as highest attained educational level, (ii) low disposable income, and (iii) manual profession in a given parish. Accounted for sex, age at diagnosis, and calendar time by design

^b^Categorized according to the highest attained level [basic (primary and lower secondary education, ≤ 9 years); medium (upper secondary including vocational upper secondary education, 10–12 years); higher (> 12 years)]

^c^Refers to the annual individual income after tax, interest and alimony payments, categorized into *low, medium, and high* based on the income quintiles of the entire Danish population by calendar year and sex (1st quintile: low, 2nd and 3rd quintiles: medium, 4th and 5th quintiles: high)

Additional analyses by smaller age strata revealed that the stronger associations seen in children diagnosed at ages 5–19 years with ependymoma, embryonal tumors, or astrocytoma and other gliomas were mostly driven by the 10–19 year olds but not solely by the older adolescents (data not shown).

Examining the association of parental level of education and income in cases with younger mothers compared to older mothers indicated the tendency that the associations found for parental education and level of income were more pronounced for children with cancer of older mothers (data not shown).

## Discussion

This nationwide register study is the first assessment of socioeconomic differences in the risk of childhood CNS tumors in Denmark and one of few worldwide. We found higher SES, when operationalized as parental education or maternal income at the individual level but not when operationalized as area-level measures, being associated with a higher risk of specific CNS tumors. Higher risks of ependymoma and embryonal CNS tumors were observed for the offspring of parents with higher level of education and higher risk of all CNS tumor types in association with higher level of maternal income. Associations were often stronger in children diagnosed at ages 5–19 years compared to children diagnosed at younger age and compared to the full sample. On the contrary, we observed little evidence for an association between neighborhood SES and risk of CNS tumors. Notably, residential area SES was not a proxy of personal SES in Denmark as demonstrated by the weak correlation between individual and neighborhood SES measures.

There may be different explanations for the associations identified for parental education and maternal income including (i) selection and information bias, (ii) under-ascertainment of cases among families of lower SES, (iii) social patterning of causative risk factors of the disease, or (iv) chance.

Our use of high-quality population-based register data with almost complete coverage, not influenced by self-reported information or non-participation, makes selection and information bias a highly unlikely explanation of our observations. The Danish Cancer Registry and Central Population Register with their excellent quality and high level of completeness [29, 30, 32] enabled analysis of virtually complete childhood cancer and control group data with minimal potential for selection bias. Annual socioeconomic information at the parish level and parental highest attained education and disposable income was obtained from Statistics Denmark precluding information bias that is often seen in self-reported data [40].

Under-diagnosis and under-ascertainment of cases have been discussed previously [19, 26, 41, 42] as potential underlying mechanism of socioeconomic differences in childhood cancer. Early symptoms of a CNS tumor are usually of unspecific nature, such as headache, nausea, or vomiting, and if access to health care services depends on the economy of a family, under-diagnosis of cases may affect those with low income. Access to health care including first-line diagnostics is, however, free of charge in Denmark. If SES affects the likelihood of being diagnosed with a CNS tumor during childhood in Denmark, more subtle mechanisms must be in play, such as parents with a higher educational level being able to communicate better with health professionals or being more persistent in efforts to find an explanation for their child’s symptoms leading to further diagnostic tests, e.g., an MRI scan of their child’s brain. In principle, such mechanisms may result in both shorter time between first symptoms and diagnosis as well as diagnosis of slowly growing benign tumors, which might have otherwise remained undetected for many years or even throughout life [43]. As slowly growing benign tumors are primarily pilocytic astrocytoma and some other low-grade gliomas, we would expect to see higher risk with higher education primarily for the group of astrocytoma and other gliomas, if this mechanism was in action. However, for that diagnostic group we observed a tendency of an inverse relationship with maternal education, which speaks against this explanation.

The associations identified for parental education and maternal income may imply a pathway through individual SES-related mediators such as environmental exposures, parental occupational exposures, dietary patterns and lifestyle, family reproductive decisions, or pregnancy-related factors [2, 3, 14]. Ionizing radiation is an established risk factor for childhood CNS tumors [2] and radon in the residence accounts for half of the ionizing radiation doze in the Danish population [44]. We would expect a higher proportion of parents of higher SES to live in one-family houses in which radon concentrations are much higher than in apartments of apartment buildings. At the same time, it has been shown that although radon enters the body via inhalation, a significant amount may reach other organs including the brain [45]. This biologically plausible explanation is, however, not supported by a previous study that found no association between radon and childhood CNS tumors in Denmark [46]. Also occupational exposure to chemicals or unhealthy lifestyle (e.g., consumption of cured meat, low intake of vegetables and fruits, tobacco smoking) are unlikely to explain our findings as those usually are more prevalent in lower SES groups. In a register study from Minnesota, the social patterning of established demographic and pregnancy-related risk factors accounted for most of the socioeconomic differences seen for maternal education and neighborhood-level SES in relation to the risk of childhood CNS tumors [24]. However, in the present study, the adjustments for family and pregnancy-related factors had no appreciable effect on the results indicating that they were not responsible mediators for our results.

We undertook multiple tests and would expect one out of 20 tests to be statistically significant by chance given the chosen 5% significance level. The 360 tests of Tables 2, 3, and 4 provided 24 statistical significant results, which is not much more than the 18 expected just by chance. However, it speaks against chance as the only explanation for our findings that 23 of the 24 have ORs above 1.00; we would expect chance to have created similar numbers of significant ORs below and above 1.00. Further, the statistically significant results are most prevalent for ependymomas and embryonal CNS tumors, but we would expect a more even distribution among CNS subtypes if chance was the (only) explanation.

The relationship between SES and childhood cancer has been most exhaustively studied for leukemia with inconsistent results across studies [22, 24–26, 41, 47, 48]. Regarding CNS tumors, the literature is much more limited and the evidence [18–28] does not provide a consistent picture. Higher SES was mostly associated with higher risk [18, 21, 23–27], particularly in studies using individual-level SES indicators [18, 21, 24–26], which corresponds to our observed risk pattern of higher risks for individual CNS tumors in association with higher level of parental education and higher level of maternal income in Denmark. Only few studies investigated individual tumor types separately but noteworthy is the positive association seen for astrocytoma and other gliomas with maternal education in the US [24, 25] and Spain [18], which was not evident in our present study. Direct cross-study comparison is however hampered by considerable differences in study design and potential for bias, SES measures used, and specific CNS tumor types analyzed. Moreover, differences in the health care system including access to health care and conditions related to SES across societies may to some extent explain discrepancies across studies. The study most comparable to ours is based on data from Norway [26], a Scandinavian country with similar health care system and population-based register infrastructure. In line with our observations from Denmark was the tendency of higher risk of ependymomas and embryonal tumors in association with higher family income, whereas contrary to the present study, no association for parental educational level was found [26]. Further research in populations, which share similar welfare systems, social structure, and the population-based register infrastructure would complement our findings and might provide a better understanding of the underlying pathways of our observations.

A significant strength of our study is the design, including both individual-level and neighborhood-level SES measures assessed at different points in time and analyzed by individual CNS tumor types and specific age groups. Given that different cancer types and subtypes likely have different etiology [2], it is crucial to assess also socioeconomic differences tumor type specifically. Our study is among the first to do so. Most previous studies considered childhood SES either at time of birth or diagnosis and rarely distinguished between different SES measures acting at different time points or evaluating potential differences between SES measures [19–23, 25, 27, 28]. We assessed the potential effect of SES during the separate stages of prenatal development and childhood and differences between individual-level and neighborhood-level SES measures as previously suggested [42]. By being able to account for demographic and pregnancy-related risk factors, we demonstrated that our observed associations with individual SES were not mediated through the social patterning of those factors.

A limitation of our study is the size of our study population, albeit unavoidable as it reflects the rarity of childhood CNS tumors and the childhood population size of Denmark. Even when including as many as 1,273 childhood CNS tumor cases, the smaller sample size for tumor type-specific analyses resulted often in imprecise effect estimates and prevented us from assessing more thoroughly the effects in more defined age groups, parental age, and by calendar period.

In conclusion, this large nationwide register study with minimal potential for bias indicated a higher risk of specific CNS tumors among children of parents with higher educational level and mothers with higher level of income. Under-ascertainment of cases among families with low income or basic education is unlikely to explain these socioeconomic differences, as Denmark is a country with free access to high-quality health care irrespective of SES and has one of the most complete cancer registries worldwide. Future research addressing explicitly the underlying mechanisms of socioeconomic differences in the risk of childhood CNS tumors in different countries may help to enhance etiologic insights of the disease occurrence.

## Electronic supplementary material

Below is the link to the electronic supplementary material.

Supplementary file1 (DOCX 26 kb) **Table S1. **Distribution of individual level socioeconomic measures among cases of CNS tumors in children aged 0–19 years born and diagnosed between 1981 and 2013 in Denmark and matched controls.

Supplementary file2 (DOCX 28 kb) **Table S2.** Distribution of neighborhood socioeconomic measures among cases of CNS tumors in children aged 0–19 years born and diagnosed between 1986 and 2013 in Denmark and matched controls.

Supplementary file3 (DOCX 17 kb) **Table S3.** Spearman’s rank correlation coefficients for maternal and paternal highest attained education and disposable income by time of assessment.

Supplementary file4 (DOCX 14 kb) **Table S4.** Spearman’s rank correlation coefficients for socioeconomic measures at time before diagnosis.

Supplementary file5 (DOCX 18 kb) **Table S5.** Association between maternal disposable income and risk of CNS tumors in children aged 5–19 years at diagnosis, by CNS tumor type and time of assessment.

## Data Availability

The data that support the findings of this study were accessed remotely on a secure platform at Statistics Denmark. Any access to data requires permission from Statistics Denmark and the Danish Cancer Society. All statistical analyses were performed remotely by accessing a secure platform at Statistics Denmark and using STATA, version 14.2.
