# Supplementary material for: Socioeconomic differences in the risk of childhood central nervous system tumors in Denmark: a nationwide register-based case–control study
Source: Cancer Causes Control. 2020 Aug 7;31(10):915–29. doi: 10.1007/s10552-020-01332-x (PMC7458950; doi:10.1007/s10552-020-01332-x)
Supplement: Supplementary file 1 — Supplementary file1 (DOCX 26 kb) Table S1. Distribution of individual level socioeconomic measures among cases of CNS tumors in children aged 0–19 years born and diagnosed between 1981 and 2013 in Denmark and matched controls. [file 10552_2020_1332_MOESM1_ESM.docx]

Cancer Causes & Control

**Socioeconomic differences in the risk of childhood central nervous system tumours in Denmark: A nationwide register-based case-control study**

*Friederike Erdmann*, Ulla Arthur Hvidtfeldt, Mette Sørensen, Ole Raaschou-Nielsen*

*Danish Cancer Society Research Center, Danish Cancer Society, Strandboulevarden 49, 2100 Copenhagen, Denmark; contact: [friederike.erdmann@uni-mainz.de](mailto:friederike.erdmann@uni-mainz.de)

**Table S1:** Distribution of individual level socioeconomic measures among cases of CNS tumours^a^ in children aged 0-19 years born and diagnosed between 1981 and 2013 in Denmark and matched controls.

|  |  | **Controls** | **All CNS tumours** | **Ependymoma** | **Astrocytoma and other gliomas** | **Embryonal CNS tumours** | **Other specified and unspecified** |
| --- | --- | --- | --- | --- | --- | --- | --- |
|  |  | **N = 5086** | **N = 1273** | **N = 99** | **N = 426** | **N = 182** | **N = 566** |
|  |  | **%** | **%** | **%** | **%** | **%** | **%** |
| **Maternal education^b^** | | | | | | | |
| ***At conception*** | Basic | **26.4** | **26.5** | **23.4** | **28.9** | **23.4** | **26.3** |
|  | Medium | **48.3** | **45.9** | **51.1** | **48.6** | **45.7** | **43.0** |
|  | High | **25.2** | **27.6** | **25.5** | **22.5** | **30.9** | **30.7** |
| ***During pregnancy*** | Basic | **25.2** | **24.9** | **22.9** | **27.9** | **20.9** | **24.2** |
|  | Medium | **47.2** | **45.0** | **44.8** | **47.2** | **46.3** | **43.0** |
|  | High | **27.6** | **30.1** | **32.3** | **24.9** | **32.8** | **32.8** |
| ***Before diagnosis*** | Basic | **21.1** | **20.2** | **18.8** | **23.1** | **16.9** | **19.0** |
|  | Medium | **47.1** | **44.7** | **44.8** | **46.6** | **46.1** | **42.8** |
|  | High | **31.8** | **35.3** | **36.5** | **30.3** | **37.1** | **38.3** |
| **Paternal education^b^** | | | | | | | |
| ***At conception*** | Basic | **22.6** | **21.8** | **14.7** | **22.1** | **24.6** | **21.9** |
|  | Medium | **55.6** | **56.4** | **67.4** | **59.2** | **49.1** | **54.7** |
|  | High | **21.8** | **21.8** | **17.9** | **18.7** | **26.3** | **23.4** |
| ***During pregnancy*** | Basic | **21.8** | **21.5** | **15.6** | **21.6** | **24.0** | **21.6** |
|  | Medium | **55.0** | **55.0** | **64.6** | **58.3** | **48.6** | **52.8** |
|  | High | **23.2** | **23.6** | **19.8** | **20.1** | **27.4** | **25.7** |
| ***Before diagnosis*** | Basic | **20.0** | **20.1** | **14.4** | **20.4** | **23.4** | **19.7** |
|  | Medium | **54.2** | **52.2** | **62.9** | **54.5** | **45.7** | **50.6** |
|  | High | **25.9** | **27.8** | **22.7** | **25.1** | **30.9** | **29.8** |
| **Maternal income^c^** | | | | | | | |
| ***At conception*** | Low | **11.0** | **9.1** | **11.2** | **8.5** | **6.7** | **10.0** |
|  | Medium | **37.7** | **37.5** | **30.6** | **39.9** | **42.8** | **35.3** |
|  | High | **51.4** | **53.4** | **58.2** | **51.7** | **50.6** | **54.7** |
| ***During pregnancy*** | Low | **9.6** | **8.2** | **10.2** | **8.2** | **6.1** | **8.5** |
|  | Medium | **39.0** | **38.0** | **35.7** | **39.2** | **43.9** | **35.6** |
|  | High | **51.4** | **53.8** | **54.1** | **52.6** | **50.0** | **55.9** |
| ***Before diagnosis*** | Low | **6.1** | **4.1** | **5.2** | **3.1** | **5.6** | **4.3** |
|  | Medium | **28.6** | **30.0** | **33.0** | **32.4** | **28.3** | **28.3** |
|  | High | **65.2** | **65.9** | **61.9** | **64.5** | **66.1** | **67.4** |
| **Paternal income^c^** | | | | | | | |
| ***At conception*** | Low | **9.8** | **9.8** | **11.2** | **11.6** | **6.7** | **9.2** |
|  | Medium | **43.4** | **46.0** | **53.1** | **44.6** | **49.4** | **44.8** |
|  | High | **46.9** | **44.2** | **35.7** | **43.8** | **43.9** | **46.0** |
| ***During pregnancy*** | Low | **9.3** | **9.9** | **9.2** | **11.4** | **6.7** | **10.0** |
|  | Medium | **42.5** | **44.4** | **53.1** | **43.4** | **45.0** | **43.4** |
|  | High | **48.2** | **45.7** | **37.8** | **45.3** | **48.3** | **46.7** |
| ***Before diagnosis*** | Low | **8.5** | **7.5** | **6.1** | **8.8** | **6.7** | **6.9** |
|  | Medium | **35.6** | **37.8** | **43.9** | **39.4** | **40.0** | **34.9** |
|  | High | **56.0** | **54.7** | **50.0** | **51.8** | **53.3** | **58.3** |

Missing information: maternal education at conception: 4.5%; maternal education during pregnancy: 2.6%; maternal education before diagnosis: 2.1%; paternal education at conception: 5.5%; paternal education during pregnancy: 4.1%; paternal education before diagnosis: 3.3%; maternal income at conception: 0.5%; maternal income during pregnancy: 0.3%; maternal income before diagnosis: 1.0%; paternal income at conception: 1.2%; paternal income during pregnancy: 1.1%; paternal income before diagnosis: 2.1%

^a^Classified by the International Classification of Childhood Cancer (ICCC), up to 2003 by Birch & Marsden (first edition) and from 2003 onwards by ICCC-3rd version. Grouped as follows: Ependymoma (defined by ICCC 1 and ICCC3 group 3a), astrocytoma and other gliomas (ICCC 1 and ICCC 3 groups 3b and 3d combined), embryonal CNS tumours (defined by ICCC 1 and ICCC3 group 3c) and other specified and unspecified (ICCC 1 and ICCC3 groups 3e and 3f combined).

^b^ Categorised according to highest attained level (basic [primary and lower secondary education, ≤9 years in Denmark]; medium [upper secondary including vocational upper secondary education, 10-12 years]; higher [>12 years])

^c^ Refers to the annual individual income after tax, interest and alimony payments, categorised into *low, medium and high* based on the income quintiles of the entire Danish population by calendar year and sex (1^st^ quintile: low, 2^nd^ and 3^rd^ quintiles: medium, 4^th^ and 5^th^ quintiles: high).
