# Supplementary material for: Socioeconomic differences in the risk of childhood central nervous system tumors in Denmark: a nationwide register-based case–control study
Source: Cancer Causes Control. 2020 Aug 7;31(10):915–29. doi: 10.1007/s10552-020-01332-x (PMC7458950; doi:10.1007/s10552-020-01332-x)
Supplement: Supplementary file 2 — Supplementary file2 (DOCX 28 kb) Table S2. Distribution of neighborhood socioeconomic measures among cases of CNS tumors in children aged 0–19 years born and diagnosed between 1986 and 2013 in Denmark and matched controls. [file 10552_2020_1332_MOESM2_ESM.docx]

Cancer Causes & Control

**Socioeconomic differences in the risk of childhood central nervous system tumours in Denmark: A nationwide register-based case-control study**

*Friederike Erdmann*, Ulla Arthur Hvidtfeldt, Mette Sørensen, Ole Raaschou-Nielsen*

*Danish Cancer Society Research Center, Danish Cancer Society, Strandboulevarden 49, 2100 Copenhagen, Denmark; contact: [friederike.erdmann@uni-mainz.de](mailto:friederike.erdmann@uni-mainz.de)

**Table S2:** Distribution of neighbourhood socioeconomic measures among cases of CNS tumours^a^ in children aged 0-19 years born and diagnosed between 1986 and 2013 in Denmark and matched controls.

|  |  | **Controls** | **All CNS tumours** | **Ependymoma** | **Astrocytoma and other gliomas** | **Embryonal CNS tumours** | **Other specified and unspecified** |
| --- | --- | --- | --- | --- | --- | --- | --- |
|  |  | **N = 4174** | **N = 1045** | **N = 81** | **N = 333** | **N = 154** | **N = 477** |
|  |  | **%** | **%** | **%** | **%** | **%** | **%** |
| **Neighbourhood basic education^b^** | | | | | | | |
| ***At conception*** | 5 *(low SES)* | **21.1** | **19.8** | **17.3** | **18.0** | **18.8** | **21.7** |
|  | 4 | **20.6** | **19.5** | **22.7** | **21.2** | **16.0** | **18.8** |
|  | 3 | **19.2** | **20.4** | **22.7** | **22.6** | **20.1** | **18.6** |
|  | 2 | **19.6** | **20.1** | **18.7** | **18.0** | **25.7** | **19.9** |
|  | 1 *(high SES)* | **19.5** | **20.4** | **18.7** | **20.3** | **19.4** | **21.0** |
| ***During pregnancy*** | 5 | **21.2** | **19.8** | **18.2** | **18.7** | **21.4** | **20.4** |
|  | 4 | **20.2** | **20.2** | **20.8** | **21.8** | **15.9** | **20.4** |
|  | 3 | **19.0** | **19.7** | **22.1** | **20.3** | **18.6** | **19.4** |
|  | 2 | **20.1** | **20.4** | **18.2** | **20.3** | **26.9** | **18.9** |
|  | 1 | **19.6** | **19.7** | **20.8** | **19.0** | **17.2** | **20.9** |
| ***Before diagnosis*** | 5 | **21.1** | **22.7** | **19.7** | **19.8** | **22.5** | **25.3** |
|  | 4 | **19.3** | **19.7** | **25.0** | **21.9** | **15.2** | **18.7** |
|  | 3 | **20.6** | **19.3** | **19.7** | **21.0** | **17.9** | **18.5** |
|  | 2 | **19.8** | **18.6** | **19.7** | **17.0** | **24.5** | **17.7** |
|  | 1 | **19.3** | **19.7** | **15.8** | **20.4** | **19.9** | **19.8** |
| **Neighbourhood low income^c^** | | | | | | | |
| ***At conception*** | 5 *(low SES)* | **25.0** | **24.6** | **13.3** | **24.8** | **27.8** | **25.2** |
|  | 4 | **20.5** | **21.7** | **28.0** | **21.2** | **18.8** | **21.9** |
|  | 3 | **19.2** | **18.9** | **22.7** | **21.2** | **18.8** | **16.8** |
|  | 2 | **18.4** | **16.5** | **18.7** | **15.0** | **12.5** | **18.4** |
|  | 1 *(high SES)* | **16.9** | **18.3** | **17.3** | **17.7** | **22.2** | **17.7** |
| ***During pregnancy*** | 5 | **24.6** | **23.6** | **9.1** | **23.1** | **27.6** | **25.2** |
|  | 4 | **20.3** | **22.3** | **24.7** | **23.4** | **18.6** | **22.4** |
|  | 3 | **19.2** | **19.5** | **27.3** | **20.9** | **20.0** | **17.2** |
|  | 2 | **19.1** | **16.6** | **18.2** | **15.0** | **11.7** | **18.9** |
|  | 1 | **16.9** | **18.0** | **20.8** | **17.8** | **22.1** | **16.3** |
| ***Before diagnosis*** | 5 | **18.9** | **18.9** | **10.5** | **20.4** | **17.2** | **19.8** |
|  | 4 | **18.3** | **20.9** | **23.7** | **19.2** | **19.9** | **21.9** |
|  | 3 | **20.8** | **20.3** | **26.3** | **21.6** | **19.9** | **18.5** |
|  | 2 | **20.4** | **18.6** | **21.1** | **16.1** | **21.2** | **19.2** |
|  | 1 | **21.6** | **21.4** | **18.4** | **22.8** | **21.9** | **20.6** |
| **Neighbourhood manual profession^d^** | | | | | | | |
| ***At conception*** | 5 *(low SES)* | **19.6** | **16.5** | **18.7** | **15.7** | **11.1** | **18.4** |
|  | 4 | **19.2** | **21.1** | **29.3** | **20.6** | **25.7** | **18.6** |
|  | 3 | **19.7** | **18.9** | **20.0** | **19.3** | **16.7** | **19.3** |
|  | 2 | **19.6** | **19.9** | **14.7** | **19.3** | **22.9** | **20.1** |
|  | 1 *(high SES)* | **21.9** | **23.6** | **17.3** | **25.2** | **23.6** | **23.7** |
| ***During pregnancy*** | 5 | **20.2** | **17.8** | **20.8** | **17.5** | **14.5** | **18.5** |
|  | 4 | **19.0** | **21.6** | **27.3** | **21.5** | **22.1** | **20.7** |
|  | 3 | **19.5** | **18.6** | **18.2** | **18.1** | **20.0** | **18.7** |
|  | 2 | **19.5** | **18.8** | **14.3** | **18.7** | **20.7** | **19.1** |
|  | 1 | **21.8** | **23.1** | **19.5** | **24.3** | **22.8** | **23.0** |
| ***Before diagnosis*** | 5 | **21.9** | **20.7** | **32.9** | **19.2** | **19.2** | **20.2** |
|  | 4 | **20.2** | **21.9** | **19.7** | **23.7** | **23.2** | **20.6** |
|  | 3 | **20.5** | **21.4** | **18.4** | **19.8** | **20.5** | **23.4** |
|  | 2 | **18.6** | **17.5** | **14.5** | **17.0** | **19.9** | **17.5** |
|  | 1 | **18.9** | **18.5** | **14.5** | **20.4** | **17.2** | **18.3** |

Missing information: at conception: 5.8%; during pregnancy: 3.2%; before diagnosis: 2.2%

^a^Classified by the International Classification of Childhood Cancer (ICCC), up to 2003 by Birch & Marsden (first edition) and from 2003 onwards by ICCC-3rd version. Grouped as follows: Ependymoma (defined by ICCC 1 and ICCC3 group 3a), astrocytoma and other gliomas (ICCC 1 and ICCC 3 groups 3b and 3d combined), embryonal CNS tumours (defined by ICCC 1 and ICCC3 group 3c) and other specified and unspecified (ICCC 1 and ICCC3 groups 3e and 3f combined).

^b^Based on the proportions of inhabitants aged 30-60 years with basic highest attained educational level in a given parish. Levels of SES are consecutively numbered.

^C^Based on the proportions of inhabitants aged 30-60 years with low disposable income (defined as family disposable income among the lowest quartile of the income distribution of the entire Danish population) in a given parish. Levels of SES are consecutively numbered.

^d^Based on the proportions of inhabitants aged 30-60 years with manual profession (defined as unskilled or semi-skilled profession) in a given parish. Levels of SES are consecutively numbered.
