# Supplementary material for: Socioeconomic differences in the risk of childhood central nervous system tumors in Denmark: a nationwide register-based case–control study
Source: Cancer Causes Control. 2020 Aug 7;31(10):915–29. doi: 10.1007/s10552-020-01332-x (PMC7458950; doi:10.1007/s10552-020-01332-x)
Supplement: Supplementary file 3 — Supplementary file3 (DOCX 17 kb) Table S3. Spearman’s rank correlation coefficients for maternal and paternal highest attained education and disposable income by time of assessment. [file 10552_2020_1332_MOESM3_ESM.docx]

Cancer Causes & Control

**Socioeconomic differences in the risk of childhood central nervous system tumours in Denmark: A nationwide register-based case-control study**

*Friederike Erdmann*, Ulla Arthur Hvidtfeldt, Mette Sørensen, Ole Raaschou-Nielsen*

*Danish Cancer Society Research Center, Danish Cancer Society, Strandboulevarden 49, 2100 Copenhagen, Denmark

Contact: [friederike.erdmann@uni-mainz.de](mailto:friederike.erdmann@uni-mainz.de)

**Table S3:** Spearman’s rank correlation coefficients for maternal and paternal highest attained education and disposable income by time of assessment

|  | **Maternal education^a^**  *at conception* | **Maternal education** *during pregnancy* | **Maternal education** *before diagnosis* | **Paternal education^a^**  *at conception* | **Paternal education**  *during pregnancy* | **Paternal education**  *before diagnosis* | **Maternal income^b^**  *at conception* | **Maternal income**  *during pregnancy* | **Maternal income** *before diagnosis* | **Paternal income^b^**  *at conception* | **Paternal income** *during pregnancy* | **Paternal income**  *before diagnosis* |
| --- | --- | --- | --- | --- | --- | --- | --- | --- | --- | --- | --- | --- |
| **Maternal education**  *at conception* | **1.0** |  |  |  |  |  |  |  |  |  |  |  |
| **Maternal education** *during pregnancy* | **0.96** | **1.0** |  |  |  |  |  |  |  |  |  |  |
| **Maternal education** *before diagnosis* | **0.86** | **0.90** | **1.0** |  |  |  |  |  |  |  |  |  |
| **Paternal education**  *at conception* |  |  |  | **1.0** |  |  |  |  |  |  |  |  |
| **Paternal education**  *during pregnancy* |  |  |  | **0.97** | **1.0** |  |  |  |  |  |  |  |
| **Paternal education**  *before diagnosis* |  |  |  | **0.91** | **0.94** | **1.0** |  |  |  |  |  |  |
| **Maternal income**  *at conception* |  |  |  |  |  |  | **1.0** |  |  |  |  |  |
| **Maternal income**  *during pregnancy* |  |  |  |  |  |  | **0.89** | **1.0** |  |  |  |  |
| **Maternal income**  *before diagnosis* |  |  |  |  |  |  | **0.50** | **0.52** | **1.0** |  |  |  |
| **Paternal income**  *at conception* |  |  |  |  |  |  |  |  |  | **1.0** |  |  |
| **Paternal income**  *during pregnancy* |  |  |  |  |  |  |  |  |  | **0.87** | **1.0** |  |
| **Paternal income**  *before diagnosis* |  |  |  |  |  |  |  |  |  | **0.51** | **0.54** | **1.0** |

^a^Categorised according to the highest attained level (basic [primary and lower secondary education, ≤9 years in Denmark]; medium [upper secondary including vocational upper secondary education, 10-12 years]; higher [>12 years])

^b^Refers to the annual individual income after tax, interest and alimony payments, based on the income quintiles of the entire Danish population by calendar year and sex.
