# Supplementary material for: Socioeconomic differences in the risk of childhood central nervous system tumors in Denmark: a nationwide register-based case–control study
Source: Cancer Causes Control. 2020 Aug 7;31(10):915–29. doi: 10.1007/s10552-020-01332-x (PMC7458950; doi:10.1007/s10552-020-01332-x)
Supplement: Supplementary file 4 — Supplementary file4 (DOCX 14 kb) Table S4. Spearman’s rank correlation coefficients for socioeconomic measures at time before diagnosis. [file 10552_2020_1332_MOESM4_ESM.docx]

Cancer Causes & Control

**Socioeconomic differences in the risk of childhood central nervous system tumours in Denmark: A nationwide register-based case-control study**

*Friederike Erdmann*, Ulla Arthur Hvidtfeldt, Mette Sørensen, Ole Raaschou-Nielsen*

*Danish Cancer Society Research Center, Danish Cancer Society, Strandboulevarden 49, 2100 Copenhagen, Denmark; contact: [friederike.erdmann@uni-mainz.de](mailto:friederike.erdmann@uni-mainz.de)

**Table S4:** Spearman’s rank correlation coefficients for socioeconomic measures at time before diagnosis.

|  | **Maternal education^a^** | **Paternal education^a^** | **Maternal income^b^** | **Paternal income^b^** | **Neighbourhood SES (% basic education)^c^** | **Neighbourhood SES (% low income)^d^** | **Neighbourhood SES (% manual profession)^e^** |
| --- | --- | --- | --- | --- | --- | --- | --- |
| **Maternal education** | **1.0** |  |  |  |  |  |  |
| **Paternal education** | **0.44** | **1.0** |  |  |  |  |  |
| **Maternal income** | **0.32** | **0.20** | **1.0** |  |  |  |  |
| **Paternal income** | **0.24** | **0.35** | **0.19** | **1.0** |  |  |  |
| **Neighbourhood SES (% basic education)** | **0.25** | **0.24** | **0.18** | **0.21** | **1.0** |  |  |
| **Neighbourhood SES (% low income)** | **0.12** | **0.11** | **0.19** | **0.21** | **0.48** | **1.0** |  |
| **Neighbourhood SES (% manual profession)** | **0.16** | **0.18** | **0.07** | **0.08** | **0.59** | **-0.11** | **1.0** |

^a^ Categorised according to the highest attained level (basic [primary and lower secondary education, ≤9 years in Denmark]; medium [upper secondary including vocational upper secondary education, 10-12 years]; higher [>12 years])

^b^ Refers to the annual individual income after tax, interest and alimony payments, based on the income quintiles of the entire Danish population by calendar year and sex.

^c^ Based on the proportions of inhabitants aged 30-60 years with basic highest attained educational level in a given parish.

^d^ Based on the proportions of inhabitants aged 30-60 years with low disposable income (defined as family disposable income among the lowest quartile of the income distribution of the entire Danish population) in a given parish.

^e^ Based on the proportions of inhabitants aged 30-60 years with manual profession (defined as unskilled or semi-skilled profession) in a given parish.
