# Supplementary material for: Socioeconomic differences in the risk of childhood central nervous system tumors in Denmark: a nationwide register-based case–control study
Source: Cancer Causes Control. 2020 Aug 7;31(10):915–29. doi: 10.1007/s10552-020-01332-x (PMC7458950; doi:10.1007/s10552-020-01332-x)
Supplement: Supplementary file 5 — Supplementary file5 (DOCX 18 kb) Table S5. Association between maternal disposable income and risk of CNS tumors in children aged 5–19 years at diagnosis, by CNS tumor type and time of assessment. [file 10552_2020_1332_MOESM5_ESM.docx]

Cancer Causes & Control

**Socioeconomic differences in the risk of childhood central nervous system tumours in Denmark: A nationwide register-based case-control study**

*Friederike Erdmann*, Ulla Arthur Hvidtfeldt, Mette Sørensen, Ole Raaschou-Nielsen*

*Danish Cancer Society Research Center, Danish Cancer Society, Strandboulevarden 49, 2100 Copenhagen, Denmark

Contact: friederike.erdmann@uni-mainz.de

| **Table S5.** Association^a^ between maternal disposable income^b^ and risk of CNS tumours in children aged 5-19 years at diagnosis, by CNS tumour type^c^ and time of assessment. | | | | | | |
| --- | --- | --- | --- | --- | --- | --- |
|  |  | **All CNS tumours** | **Ependymoma** | **Astrocytoma and other gliomas** | **Embryonal CNS tumours** | **Other specified and unspecified** |
|  |  | **OR (95% CI)** | **OR (95% CI)** | **OR (95% CI)** | **OR (95% CI)** | **OR (95% CI)** |
| ***At conception*** | Low | **1.0** | **1.0** | **1.0** | **1.0** | **1.0** |
|  | Medium | **1.34 (0.99-1.81)** | **3.59 (0.42-30.55)** | **1.37 (0.80-2.37)** | **2.07 (0.75-5.75)** | **1.17 (0.79-1.74)** |
|  | High | **1.49 (1.10-2.00)** | **6.48 (0.82-51.31)** | **1.38 (0.80-2.37)** | **2.34 (0.82-6.69)** | **1.31 (0.88-1.94)** |
| ***During pregnancy*** | Low | **1.0** | **1.0** | **1.0** | **1.0** | **1.0** |
|  | Medium | **1.28 (0.94-1.74)** | **4.18 (0.48-36.76)** | **1.01 (0.60-1.72)** | **2.75 (0.90-8.40)** | **1.20 (0.78-1.83)** |
|  | High | **1.42 (1.04-1.94)** | **5.60 (0.70-45.07)** | **1.06 (0.63-1.78)** | **2.35 (0.75-7.33)** | **1.46 (0.95-2.23)** |
| ***Before diagnosis*** | Low | **1.0** | **1.0** | **1.0** | **1.0** | **1.0** |
|  | Medium | **1.59 (1.00-2.54)** | **1.15 (0.11-12.07)** | **2.56 (1.06-6.21)** | **0.77 (0.24-2.53)** | **1.42 (0.74-2.72)** |
|  | High | **1.63 (1.04-2.55)** | **2.68 (0.30-23.90)** | **2.10 (0.89-4.99)** | **1.79 (0.60-5.34)** | **1.30 (0.70-2.43)** |

^a^Conditional logistic regression analyses [odds ratio (and 95% confidence interval)] adjusted for maternal or paternal age respectively, modelled as continuous variable.

^b^ Refers to the annual individual income after tax, interest and alimony payments, categorised into *low, medium and high* based on the income quintiles of the entire Danish population by calendar year and sex (1^st^ quintile: low, 2^nd^ and 3^rd^ quintiles: medium, 4^th^ and 5^th^ quintiles: high)

^c^Classified by the International Classification of Childhood Cancer, up to 2003 by Birch & Marsden (first edition) and from 2003 onwards by ICCC-3. Grouped as follows: Ependymoma (defined by ICCC 1 and ICCC3 group 3a), astrocytoma and other gliomas (ICCC 1 and ICCC 3 groups 3b and 3d combined), embryonal CNS tumours (defined by ICCC 1 and ICCC3 group 3c) and other specified and unspecified (ICCC 1 and ICCC3 groups 3e and 3f combined).
